# Early graft loss due to acute thrombotic microangiopathy accompanied by complement gene variants in living-related kidney transplantation: case series report

**DOI:** 10.1186/s12882-022-02868-7

**Published:** 2022-07-14

**Authors:** Qianqian Wu, Xiaohui Tian, Nianqiao Gong, Jin Zheng, Dandan Liang, Xue Li, Xia Lu, Wujun Xue, Puxun Tian, Jiqiu Wen

**Affiliations:** 1 National Clinical Research Center of Kidney Diseases, Jinling Hospital, Medical School of Nanjing University, Nanjing, Jiangsu China; 2 Department of Kidney Transplantation, Nephropathy Hospital, The First Affiliated Hospital of Xi’an Jiaotong University, Xi’an, Shanxi China; 3 Institute of Organ Transplantation, Tongji Hospital, Tongji Medical College, Huazhong University of Science and Technology, Wuhan, Hubei China

**Keywords:** Thrombotic microangiopathy, Complement factor H, Gene variant, Living donor, Kidney transplantation

## Abstract

**Background:**

Recently, early graft loss has become very rare in living-related kidney transplantation (LKT) as a result of decreased risk of hyperacute rejection and improvements in immunosuppressive regimens. Post-transplant acute thrombotic microangiopathy (TMA) is a rare, multi-factorial disease that often occurs shortly after kidney transplantation and is usually resistant to treatment with dismal renal outcomes. The complement genetic variants may accelerate the development of TMA. However, the complement genetic test was seldom performed in unknown native kidney disease recipients scheduled for LKT.

**Case presentation:**

We reported three cases of unknown native kidney diseases who had fulminant TMA in the allograft shortly after LKT. Both the donors and the recipients were noted to carry complement genetic variants, which were identified by genetic testing after transplantation. However, all recipients were refractory to treatment and had allograft loss within 3 months after LKT.

**Conclusion:**

This case series highlights the suggestion to screen complement gene variants in both the donors and the recipients with unknown native kidney diseases scheduled for LKT.

**Supplementary Information:**

The online version contains supplementary material available at 10.1186/s12882-022-02868-7.

## Background

Currently, owing to improved screening regimens for transplant candidates and better immunosuppression, the short-term prognosis of kidney transplantation has greatly improved [1, 2], only 0.4% of patients had graft loss due to severe acute rejection during the first post-transplant year [2]. However, vascular thrombosis has become a common reason for early graft loss [2, 3]. TMA is a rare disease that is clinically characterized by hemolytic microangiopathic anemia, thrombocytopenia, and organ injuries due to the presence of thrombi in the capillaries and small arteries. Post-transplant TMA is relatively uncommon in the graft biopsy, with de novo and recurrent TMA being discovered in 0.8%-14% [4, 5] and 9%-29.4% [5, 6] of patients, respectively. Noteworthy, complement regulatory genetic variants have been observed to accelerate the development of TMA in renal allografts [7]. Patients with complement genetic variants had a higher risk of recurrence of TMA, whereas the highest risk of graft loss was observed in patients with both complement pathway variants and low C3 [8]. Le Quintrec et al. found that 7 out of 24 de novo TMA (29%) had complement factor H (CFH) or complement factor I (CFI) gene variants, two of whom had acute rejection and calcineurin inhibitor (CNI) toxicity, respectively [7]. In 2018, we reported a kidney recipient of concomitant C3 glomerulonephritis and TMA failed to respond to plasma exchange and had early graft loss who had two CFI genetic variations and low serum C3 level [9]. These two CFI genetic variants were not verified as pathogenic genes for TMA in a subsequent study with the CRISPR/Cas9 system to make mutant mouse lines that carried D288G and P467S variants in CFI in the mouse model [10].

Here we report 3 cases of fulminant TMA shortly after LKT accompanied by complement gene variants in donor-recipient pairs, leading to allograft loss within 3 months post-transplant in all cases.

## Case presentation

### Patient 1

The first patient was a 23-year-old man who received LKT from his mother for end-stage renal disease (ESRD) with unknown native kidney disease. After transplantation, the serum creatinine (Scr) decreased to reach a nadir of 195 μmol/L. On post-operative day (POD) 13, Scr increased to 295 μmol/L. He was treated with pulse methylprednisolone (500 mg/d for 3 days) considering acute cellular rejection. After treatment, the Scr decreased to 228 μmol/L on POD 18, and the patient was discharged. The clinical data are listed in Table 1.

**Table 1:** Clinical characteristics and outcomes

**At Tx**	**Case 1**	**Case 2**	**Case 3**
Age at Tx	23	24	36
Cause of ESRD	Unknown	Unknown	Unknown
Duration of dialysis, mos	16	9	10
HLA allele mismatches	4/8	3/8	4/8
Blood type (Recipient & Donor)	O RhD +	A RhD +	O RhD +
Donor age at Tx	48	44	58
Donor type	LKT	LKT	LKT
CDC test	Negative	Negative	Negative
PRA pre-Tx			
Class I	Positive (NDSA)	Negative	Negative
Class II	Positive (NDSA)	Negative	Negative
Induction therapy	ATG	CTX	ATG
Immunosuppressive therapy	TAC + MMF + PED	TAC + MMF + PED	CsA + MMF + PED
Adverse events after Tx	Acute rejection	No	No
SCr at discharge (μmol/L)	228	95	110
**At diagnosis**	**Case 1**	**Case 2**	**Case 3**
Time to onset post-Tx, days	45	60	39
Time to diagnosis post-Tx, days	64	64	68
Proteinuria	±	2 +	3 +
Scr (μmol/L)	840	300	605
LDH (U/L)	742	463	612
HB (g/L)	81	67	94
PLT (× 109/L)	84	121	300
PRA	Negative	Negative	Positive (NDSA)
Serum C3 (g/L)	NA	0.90	0.48
Schistocytes on a PBS	< 2%	NA	< 2%
Serum anti-GBM antibody	Negative	Negative	Negative
Serum ANCA	Negative	Negative	Negative
TAC blood concentration (ng/ml)	7.7	9.9	7.2
Immunosuppressive therapy	TAC + MMF + PED	TAC + MMF + PED	CsA + MMF + PED
Concomitant events	ABMR? CMV infection	CNI toxicity	ABMR
Genetic testing (Recipient & Donor)	Variant c.721C > T in CFHR3 gene	Variant c.3572C > T in CFH gene	Variant c.3578C > G in CFH gene
**Treatment and prognosis**	**Case 1**	**Case 2**	**Case 3**
Treatment therapy after diagnosis (time)	Ganciclovir PE (POD70, 72, 76) CRRT(POD78, 83, 86)	Convert TAC to SRL	PE Bortezomib (POD73, 78) Rituximab (POD75)
Graft survival from time of Tx, days	66	84	54
Outcome	Dialysis	Dialysis	Dialysis

*Tx* Transplantation, *ESRD* End-stage renal disease, *mos* Months, *HLA* Human lymphocyte antigen, *LKT* living-related kidney transplantation, *CDC* Complement-dependent cytotoxicity, *PRA* Panel-reactive antibody, *NDSA* Nondonor-specific antibodies, *ATG* Antithymocyte globulin, *CTX* Cyclophosphamide, *TAC* Tacrolimus, *MMF* Mycophenolate mofetil, *PED* Prednisone, *SCr* serum creatinine, *HB* Hemoglobin, *PLT* Platelets, *CsA* Cyclosporine A, *CMV* Cytomegalovirus, *CNI* Calcineurin inhibitor, *PBS* Peripheral blood smear, *GBM* Glomerular basement membrane, *ANCA* Antineutrophil cytoplasmic antibodies, *PLA2R* Anti-phospholipase A2 receptor, *ABMR* Antibody-mediated rejection, *PE* Plasma exchange, *CRRT* Continuous renal replacement therapy, *SRL* Sirolimus, *POD* Postoperative day

On POD 45, the patient was re-admitted with sudden anuresis (30 ml/d) and fever (39℃) on the second day after removing the double-J stent (DJS). On the day of admission, renal transplant ultrasound was performed and showed an increased arterial resistance index (RI) of the transplant kidney (renal graft aorta RI: 0.82). His Scr rose from 185 μmol/L to 260 μmol/L. DJS was re-inserted the next day (POD 46) for suspected acute complete obstruction according to the course of the disease and the ultrasound findings. However, there was no significant improvement in urine volume (250 ml/d, POD 47). As the arterial RI of the transplant kidney was significantly increased (renal graft aorta RI: 0.91), he then was treated with anti-thymocyte globulin (ATG) (50 mg/d for 3 days) and methylprednisolone (500 mg/d for 3 days) suspecting of acute rejection. On POD 51, Scr still rose to 708 μmol/L despite an increase in urine volume (1310 ml/d). Next-generation sequencing testing identified cytomegalovirus (CMV) infection on POD 55. On POD 64, Scr rose to 840 μmol/L, and a renal allograft biopsy was performed.

**Fig. 1:**
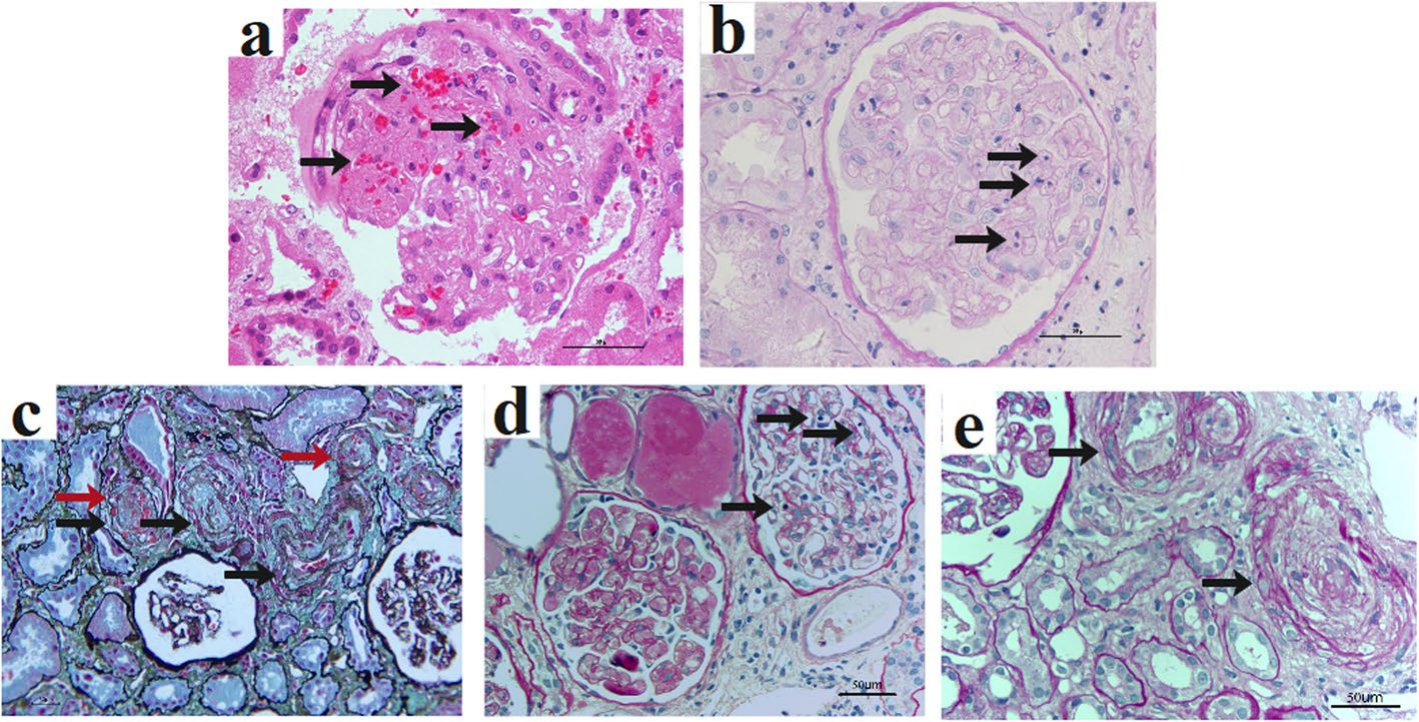
Pathological findings of three cases. **a** Case 1: mesangiolysis, and fragmented red blood cells (arrow) in the glomeruli (HE × 400). **b** Case 1: glomerular endothelial cell swelling and inflammatory cell (arrow) infiltration (PAS × 400). **c** Case 2: thickening of walls and narrowing of the lumen of arteriole (black arrow) with fragmented red blood cells (red arrow) (PASM × 200). **d** Case 3: the first allograft biopsy showed glomerular inflammatory cell (arrow) infiltration (PAS × 200). **e** Case 3: the second graft biopsy appeared an "Onion skin" pattern lesion (arrow) in the arteriole (PAS × 200)

Light microscopy (equipment, Nikon ECLIPSE 80i; software, NIS-Elementary TS BR 3.2) on transplant biopsy revealed glomerulitis, acute tubulointerstitial nephritis (ATIN), and typical TMA. In addition, fragmented red blood cells in the glomerular capillary loops and interstitial hemorrhage were noted (summarized in Fig. 1 and Table 2). Genetic testing (Supplementary Information) of the recipient revealed a homozygous variant (c.721C>T, p.P241S) in the complement factor H related protein 3 (CFHR3) gene, while the donor had a heterozygous variant in the same gene. The patient received PE (plasma exchange) therapy three times (Table 1). Nonetheless, there was no significant improvement in Scr, and the patient returned to hemodialysis.

**Table 2:** Biopsy findings of three cases

Parameter	**Case 1**	**Case 2**	**Case 3**		
			**1st**	**2nd**	**3rd**
Post-Tx, days	64	64	48	68	88
Number of glomerulus, n	9	23	11	31	6
LM					
Glomerulus	Mesangiolysis Shrunken capillary loops Glomerular inflammatory cell infiltration	Shrunken capillary loops Segmental capillary wall double contours	Segmental glomerular capillary fibrinoid necrosis Glomerular inflammatory cell infiltration (g2)		
Tubulointerstitium	Interstitial hemorrhage ATIN	Tubular atrophy and interstitial fibrosis ATN	No	Tubular atrophy (ct1)	Tubular atrophy(ct2) interstitial fibrosis(ci1)
Renal arteriole/artery	Arterial endothelial edema	Arteriolar intimal edema and thickening Arteriolar lumen occlusion	Arteriolar wall thickening	Arteriolar intimal thickening Arteriolar lumen occlusion Endoarteritis (v2)	Arteriolar wall thickening Arteriolar intimal thickening Arteriolar lumen occlusion
Thrombi location	No	No	No	Glomerular	Glomerular, PTA
Fragmented red blood cells location	Glomerular capillary loops	Arteriolar Glomerular capillary loops	No	Glomerular capillary loops	Glomerular capillary loops
IF	IgA + + , IgM +	Negative	NA	NA	NA
C4d	Negative	NA	Positive	Positive	Negative
Pathological diagnosis	TMA, ATIN, ABMR?	TMA, ATN	ABMR	ABMR, TMA	ABMR, TMA

*Tx* Transplantation, *n* Number, *PTA* Peritubular capillary, *ATIN* Acute tubulointerstitial nephritis, *ATN* Acute tubular necrosis, *NA* Not available, *TMA* Thrombotic microangiopathy, *ABMR* Antibody mediated rejection

### Patient 2

The second patient was a 24-year-old man who received LKT from his mother for an unidentified cause of ESRD (Table 1). Scr decreased to 95 μmol/L within a week after transplantation. Two months post-transplant, the patient was referred to the hospital for a Scr of 300 μmol/L. He was negative for BK virus and parvovirus B19. Renal allograft biopsy showed TMA and acute tubular necrosis (ATN) (summarized in Table 2 and Fig. 1). Besides, tubular epithelial cell vacuolization and hyaline droplet degeneration in the adventitia of arterioles were also observed. Genetic testing revealed a heterozygous variant (c.3572C > T, p.S1191L) of the CFH gene in both the patient and the donor (his mother).

On POD 68, the immunosuppressive regimen was converted from tacrolimus to sirolimus. However, the graft function did not improve. On POD 83, Scr increased to 786 μmol/L, and the patient was started on regular hemodialysis again.

### Patient 3

The third patient was a 36-year-old man who received LKT from his father for an unidentified cause of ESRD (Table 1). On POD 19, the patient was discharged with a Scr level of 110 μmol/L.

On POD 40, he was re-admitted for proteinuria (1.52 g/24 h) and elevated Scr (220 μmol/L). Then the patient was treated with methylprednisolone (500 mg/d) and cyclophosphamide (0.1 g/d) impulse therapy for 3 days. Allograft biopsy (POD 48) showed morphologic changes of acute antibody-mediated rejection (ABMR), including glomerulitis, glomerular capillary fibrinoid necrosis, thickened arterioles wall, and positive staining for C4d in peritubular capillaries (summarized in Fig. 1 and Table 2). The patient was treated with ATG and bortezomib. However, Scr still increased to 465 μmol/L. The patient began maintenance hemodialysis on POD56. PE (POD56, POD58), intravenous immunoglobulin (POD56, POD58), and bortezomib once a week (POD57) were then administered. On POD 68, the second biopsy revealed micro-thrombi in the glomerular capillary loops, endarteritis, arteriolar intima thickening, and lumen occlusion, indicating the presence of post-transplant TMA (summarized in Fig. 1 and Table 2). Regrettably, Scr continued to rise to 890 μmol/L on POD 70. Then, the patient was treated with PE, bortezomib, rituximab, and regular dialysis treatment (Table 2). During the third biopsy on POD 88, TMA and aggravated chronic renal allograft injuries were observed.

Genetic testing revealed that the patient had a heterozygous variant (c.3578C > G, p.T1193R) in the CFH gene, while the donor had the same heterozygous variant in the CFH gene.

## Discussion and conclusion

The three cases presented in this case series all share the following similarities: (1) all the recipients who had unknown native kidney diseases received LKT from one of their parents, and both the recipients and the donors had genetic variants in the complement factors; (2) all cases were identified TMA in the renal allograft by biopsy; (3) the prognosis of these cases was poor, patients had allograft loss within 3 months post-transplant.

Multiple etiologies have been identified to trigger post-transplant TMA, including ABMR, CNI toxicity, viral infections, sepsis, pregnancy, malignancies, and surgery [11]. In our case series, ABMR cannot be ruled out as a trigger for TMA in case 1. Besides, next-generation sequencing confirmed that the patient had a CMV infection. Of note, previous studies have shown that CMV infection was associated with post-transplant TMA [12–14]. CMV infection can cause endothelial cell injury indirectly and induce platelet adherence and von willebrand factor expression [15, 16]. Therefore, ABMR and CMV infection might act synergistically to cause TMA in the first case. The histological findings of case 2 showed tubular epithelial cell vacuolization and hyaline droplet degeneration in the adventitia of arterioles, suggesting that TMA may be induced by acute CNI toxicity. However, there was no improvement in Scr after conversion from tacrolimus to sirolimus. The first biopsy of case 3 showed typical ABMR [17], it was impossible to rule out the possibility of TMA caused by ABMR. Besides, cyclophosphamide metabolites are considered to cause direct endothelial capillary damage, inducing the cascade of thrombosis [18].

**Fig. 2:**
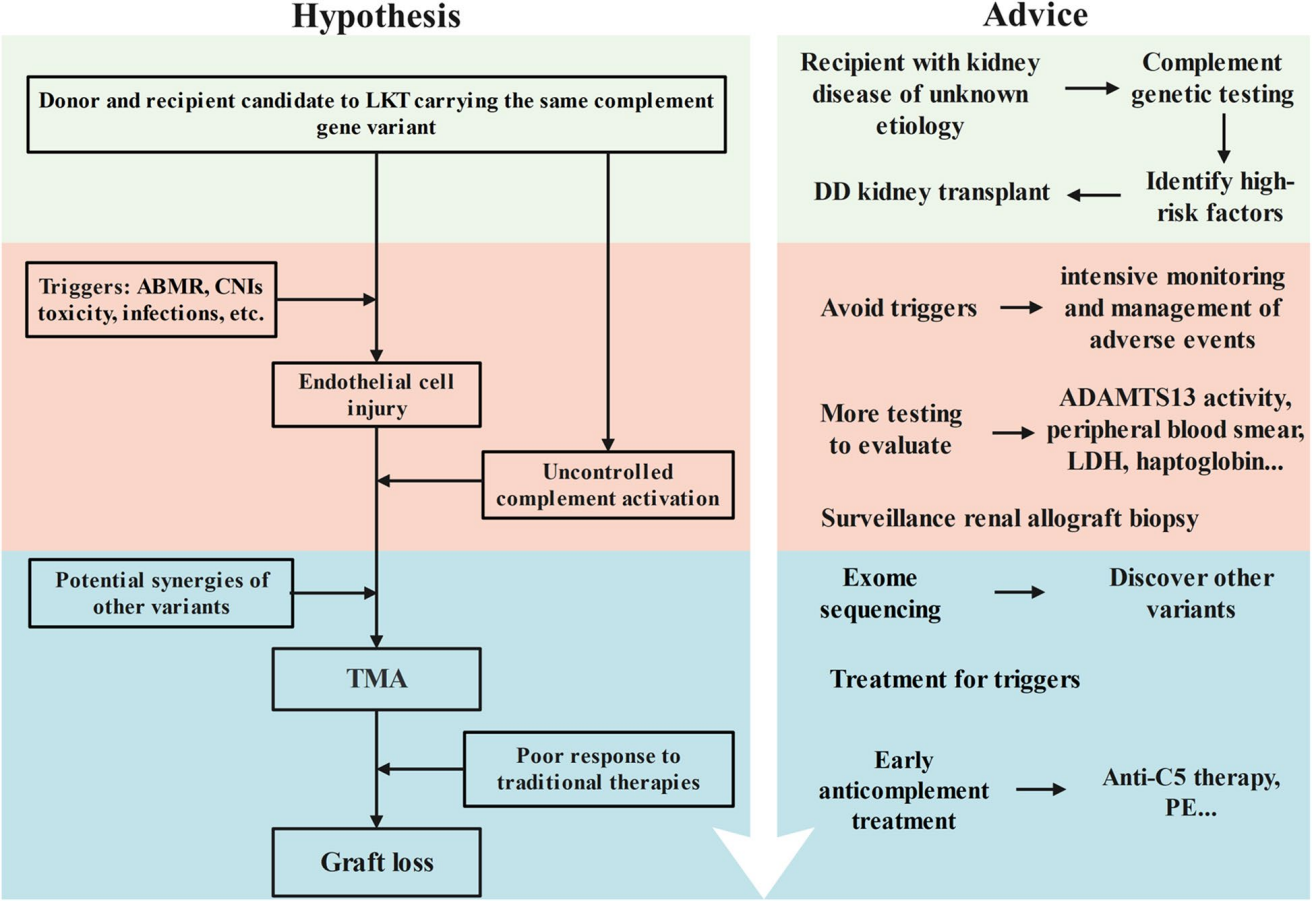
Hypothesis based on our case series: both recipients and donors carried the complement genetic abnormality, contributing to the continuous injury of endothelial cells and leading to early graft loss after activation of TMA by triggers in our case series. Advice for these patients: (1) before transplantation, recipients with kidney disease of unknown etiology may consider complement genetic testing. A deceased donor kidney transplant should be recommended if testing reveals complement genetic variant in both donor and recipient; (2) after transplantation, intensive monitoring and timely treatment of triggers are critical. Besides, surveillance allograft biopsy, more testing, and exome sequence may help to diagnosis; (3) since diagnosis of post-transplant TMA, early anti-complement treatment is necessary. LKT, living-relative kidney disease; DD, deceased donor; ABMR, antibody-mediated rejection; CNIs, calcineurin inhibitors; TMA, thrombotic microangiopathy; LDH, lactic dehydrogenase; PE, plasma exchange

The interesting part of the current study is the observation of complement gene variants in both the donors and the recipients, which may lead to complement over-activation, potentially promoting TMA. The “multiple-hit hypothesis” for TMA argues that the combination of genetic predisposition and several trigger conditions work synergistically to provoke TMA in the allograft [19]. Genetic variants or acquired abnormality in CFH could induce uncontrolled complement activation amplifying. According to the American College of Medical Genetics and Genomics guideline classification [20], CFHR3 c.721C > T (p.P241S) is a variant of benign (BA1, BS1, BP4), CFH c.3572C > T (p.S1191L) is a likely pathogenic variant (PM1, PM2, PM5, BP4) and CFH c.3578C > G (p.T1193R) is evaluated as uncertain significance (PM1, PM2, BP4). Besides, all donors who carried the same variant of complement genes as the recipients were free of kidney disease. We suspected that external factors may be required to trigger TMA, and other undiscovered genetic variants of the recipients also contribute to the disease (Fig. 2). For example, patient 1 also had a heterozygous variant in the PROS1 gene, which has been reported to be associated with protein s deficiency and would increase the risk of thrombosis [21]. Therefore, the deficiency of triggers and absence of other potential deleterious genetic variants may have led to the different clinical manifestations.

The 2015 Kidney Disease: Improve Global Outcome (KDIGO) recommends that atypical hemolytic uremic syndrome (aHUS) recipients with identified genetic or acquired factors can only be considered for LKT from donors without these factors [22]. Patients with suspected aHUS are recommended to perform genetic testing in KDIGO 2021 guideline [23]. It has been suggested that genetic testing could reveal underlying conditions in patients with an unidentified cause of ESRD pre-transplant and help in improving pre- and post-transplant management [24–26]. However, no studies have concluded that a complement genetic test is required for recipients with unknown primary disease before kidney transplantation. In our case series, recipients and donors both carried the complement genetic abnormality, which may contribute to the continuous injury of endothelial cells. Noteworthy, no abnormality was found in the routine screening program of recipients and donors, which led to the negligence of genetic testing pre-transplant. Therefore, for recipients with unknown causes of ESRD, appropriate complement genetic screening may be considered to identify potential risks with the consent of patients. Meanwhile, nephrologists should select the most suitable genetic test for the recipient [27]. The minimum set of genes that should be screened for complement genes includes CFH, CD46, CFI, CFB, THBD, CFHR1, CFHR5, and DGKE [22].

The prognosis of TMA in renal allografts is quite poor. Graft loss within 2 years of diagnosis was reported to occur in about 40% of cases, whereas 50% of patients died within 3 years after diagnosis [5]. Early anti-complement treatment is crucial to rescue renal function and avoid sequelae [22, 28]. Currently, eculizumab is the first-line effective therapy for the treatment and prevention of recurrent TMA [29–32]. Pre-emptive eculizumab treatment is sufficient to prevent the recurrence of aHUS and to maintain long-term graft function in patients with complement genetic variants [33].

However, eculizumab is not approved by the State Food and Drug Administration in China. In this case, PE was recommended by the KDIGO workshop [22], which has been found to improve graft survival by removing platelet-aggregating factors and replenishing deficient factors [12, 34]. In our case series, case 1 and case 2 received PE, while case 2 was adjusted for immunosuppression since conversion from cyclosporine to tacrolimus is the preferred therapy for cyclosporine-associated TMA [4]. For CMV infection-related TMA, intravenous ganciclovir was noted to be effective in several case reports [12, 15]. However, all graft loss occurred within 3 months post-transplant. Such rapid progression of TMA may be associated with the lack of early use of eculizumab.

Our study has some limitations. Although the recipients carried the CFH gene variants, some tests such as CFH antibody, serum CFH level, ADAMTS13 activity, haptoglobin, and Shiga toxin testing were not performed. Our limited data are insufficient to recommend widespread testing for complement variants in recipients and donors scheduled for LKT. The hypothesis based on our case series requires further animal experiments to validate.

In conclusion, we reported 3 cases that had early graft loss due to fulminant TMA accompanied by complement gene variants in both donors and recipients. Simultaneous carriage of complement genetic variants by both the donors and recipients may increase the risk of TMA after LKT. Therefore, genetic testing of the complement pathway may be considered for selected patients with unknown causes of ESRD who are scheduled for LKT.

## Supplementary Information

**Additional file 1. **Detailed genetic testing methods.

**Additional file 2: Supplementary Table 1. **Genes which were sequenced in the study.

## Data Availability

The datasets used and/or analyzed during the current study are available from the corresponding author on reasonable request.

## Abbreviations

TMA: Thrombotic microangiopathy
ABMR: Antibody-mediated rejection
CFH: Complement Factor H
CFI: Complement Factor I
LKT: Living-related kidney transplantation
ESRD: End-stage renal disease
ATG: Anti-thymocyte globulin
CMV: Cytomegalovirus
ATIN: Acute tubulointerstitial nephritis
ATN: Acute tubular necrosis
Scr: Serum creatinine
POD: Post-operative day
DJS: Double-J stent
RI: Resistance index
PE: Plasma exchange
CFHR3: Complement factor H related protein 3
KDIGO: Kidney disease: improve global outcome
aHUS: Atypical hemolytic uremic syndrome

## References

[1] Matas AJ, Payne WD, Sutherland DE, Humar A, Gruessner RW, Kandaswamy R (2001). 2,500 living donor kidney transplants: a single-center experience. Ann Surg.

[2] Helantera I, Raiha J, Finne P, Lempinen M (2018). Early failure of kidney transplants in the current era-a national cohort study. Transpl Int.

[3] Keller AK, Jorgensen TM, Jespersen B (2012). Identification of risk factors for vascular thrombosis may reduce early renal graft loss: a review of recent literature. J Transplant.

[4] Zarifian A, Meleg-Smith S, O'Donovan R, Tesi RJ, Batuman V (1999). Cyclosporine-associated thrombotic microangiopathy in renal allografts. Kidney Int.

[5] Reynolds JC, Agodoa LY, Yuan CM, Abbott KC (2003). Thrombotic microangiopathy after renal transplantation in the United States. Am J Kidney Dis.

[6] SaikumarDoradla LP, Lal H, Kaul A, Bhaduaria D, Jain M, Prasad N (2020). Clinical profile and outcomes of De novo posttransplant thrombotic microangiopathy. Saudi J Kidney Dis Transpl.

[7] Le Quintrec M, Lionet A, Kamar N, Karras A, Barbier S, Buchler M (2008). Complement mutation-associated de novo thrombotic microangiopathy following kidney transplantation. Am J Transplant.

[8] Le Quintrec M, Zuber J, Moulin B, Kamar N, Jablonski M, Lionet A (2013). Complement genes strongly predict recurrence and graft outcome in adult renal transplant recipients with atypical hemolytic and uremic syndrome. Am J Transplant.

[9] Wen J, Wang W, Xu F, Sun J, Chen J, Ni X (2018). C3 glomerulonephritis and thrombotic microangiopathy of renal allograft after pulmonary infection in a male with concomitant two complement factor I gene variations: a case report. BMC Nephrol.

[10] Song H, Zhang M, Li X, Xu F, Zhang D, Zhu X (2021). Generation and Characterization of Mouse Models of C3 Glomerulonephritis With CFI D288G and P467S Mutations. Front Physiol.

[11] Broecker V, Bardsley V, Torpey N, Perera R, Montero R, Dorling A (2019). Clinical-pathological correlations in post-transplant thrombotic microangiopathy. Histopathology.

[12] Olie KH, Goodship TH, Verlaak R, Florquin S, Groothoff JW, Strain L (2005). Posttransplantation cytomegalovirus-induced recurrence of atypical hemolytic uremic syndrome associated with a factor H mutation: successful treatment with intensive plasma exchanges and ganciclovir. Am J Kidney Dis.

[13] De Keyzer K, Van Laecke S, Peeters P, Vanholder R (2010). De novo thrombotic microangiopathy induced by cytomegalovirus infection leading to renal allograft loss. Am J Nephrol.

[14] Java A, Edwards A, Rossi A, Pandey R, Gaut J, Delos Santos R (2015). Cytomegalovirus-induced thrombotic microangiopathy after renal transplant successfully treated with eculizumab: case report and review of the literature. Transpl Int.

[15] Waiser J, Budde K, Rudolph B, Ortner MA, Neumayer HH (1999). De novo hemolytic uremic syndrome postrenal transplant after cytomegalovirus infection. Am J Kidney Dis.

[16] Rahbar A, Soderberg-Naucler C (2005). Human cytomegalovirus infection of endothelial cells triggers platelet adhesion and aggregation. J Virol.

[17] Loupy A, Haas M, Roufosse C, Naesens M, Adam B, Afrouzian M (2020). The Banff 2019 Kidney Meeting Report (I): Updates on and clarification of criteria for T cell- and antibody-mediated rejection. Am J Transplant.

[18] Iqubal A, Iqubal MK, Sharma S, Ansari MA, Najmi AK, Ali SM (2019). Molecular mechanism involved in cyclophosphamide-induced cardiotoxicity: Old drug with a new vision. Life Sci.

[19] Riedl M, Fakhouri F, Le Quintrec M, Noone DG, Jungraithmayr TC, Fremeaux-Bacchi V (2014). Spectrum of complement-mediated thrombotic microangiopathies: pathogenetic insights identifying novel treatment approaches. Semin Thromb Hemost.

[20] Richards S, Aziz N, Bale S, Bick D, Das S, Gastier-Foster J (2015). Standards and guidelines for the interpretation of sequence variants: a joint consensus recommendation of the American College of Medical Genetics and Genomics and the Association for Molecular Pathology. Genet Med.

[21] Tang L, Jian XR, Hamasaki N, Guo T, Wang HF, Lu X (2013). Molecular basis of protein S deficiency in China. Am J Hematol.

[22] Goodship TH, Cook HT, Fakhouri F, Fervenza FC, Fremeaux-Bacchi V, Kavanagh D (2017). Atypical hemolytic uremic syndrome and C3 glomerulopathy: conclusions from a "Kidney Disease: Improving Global Outcomes" (KDIGO) Controversies Conference. Kidney Int.

[23] Chadban SJ, Ahn C, Axelrod DA, Foster BJ, Kasiske BL, Kher V (2020). KDIGO Clinical Practice Guideline on the Evaluation and Management of Candidates for Kidney Transplantation. Transplantation.

[24] Ottlewski I, Munch J, Wagner T, Schonauer R, Bachmann A, Weimann A (2019). Value of renal gene panel diagnostics in adults waiting for kidney transplantation due to undetermined end-stage renal disease. Kidney Int.

[25] Mansilla MA, Sompallae RR, Nishimura CJ, Kwitek AE, Kimble MJ, Freese ME (2021). Targeted broad-based genetic testing by next-generation sequencing informs diagnosis and facilitates management in patients with kidney diseases. Nephrol Dial Transplant.

[26] Groopman EE, Marasa M, Cameron-Christie S, Petrovski S, Aggarwal VS, Milo-Rasouly H (2019). Diagnostic Utility of Exome Sequencing for Kidney Disease. N Engl J Med.

[27] Cocchi E, Nestor JG, Gharavi AG (2020). Clinical Genetic Screening in Adult Patients with Kidney Disease. Clin J Am Soc Nephrol.

[28] Legendre CM, Licht C, Muus P, Greenbaum LA, Babu S, Bedrosian C (2013). Terminal complement inhibitor eculizumab in atypical hemolytic-uremic syndrome. N Engl J Med.

[29] Johnson CK, Leca N (2015). Eculizumab use in kidney transplantation. Curr Opin Organ Transplant.

[30] Zuber J, Frimat M, Caillard S, Kamar N, Gatault P, Petitprez F (2019). Use of Highly Individualized Complement Blockade Has Revolutionized Clinical Outcomes after Kidney Transplantation and Renal Epidemiology of Atypical Hemolytic Uremic Syndrome. J Am Soc Nephrol.

[31] Avila A, Gavela E, Sancho A (2021). Thrombotic Microangiopathy After Kidney Transplantation: An Underdiagnosed and Potentially Reversible Entity. Front Med (Lausanne).

[32] Cavero T, Rabasco C, Lopez A, Roman E, Avila A, Sevillano A (2017). Eculizumab in secondary atypical haemolytic uraemic syndrome. Nephrol Dial Transplant.

[33] Krid S, Roumenina LT, Beury D, Charbit M, Boyer O, Fremeaux-Bacchi V (2012). Renal transplantation under prophylactic eculizumab in atypical hemolytic uremic syndrome with CFH/CFHR1 hybrid protein. Am J Transplant.

[34] Karthikeyan V, Parasuraman R, Shah V, Vera E, Venkat KK (2003). Outcome of plasma exchange therapy in thrombotic microangiopathy after renal transplantation. Am J Transplant.

