# Supplementary material for: Early graft loss due to acute thrombotic microangiopathy accompanied by complement gene variants in living-related kidney transplantation: case series report
Source: BMC Nephrol. 2022 Jul 14;23:249. doi: 10.1186/s12882-022-02868-7 (PMC9284761; doi:10.1186/s12882-022-02868-7)
Supplement: Supplementary file 1 — Additional file 1. Detailed genetic testing methods. [file 12882_2022_2868_MOESM1_ESM.doc]

**Additional file 1:** Detailed genetic testing methods.

**Genetic testing methods**

Genomic DNA from family members (father and mother) and patient was extracted from peripheral blood leukocytes using the DNeasy kit (Qiagen), according to the manufacturer’s instructions. Whole exomes were captured (MyGenostics Inc., Beijing, China) and sequenced on Illumina NovaSeq 6000 series sequencer (PE150). Quality control (QC) filters were applied to remove reads with low quality. Bioinformatics analysis was performed using an in-house pipeline that included genome alignment (human reference genome hg19, NCBI) with the Burrows-Wheeler Aligner (BWAMEM). We used the online system independently developed by Chigene (www.chigene.org) to annotate databasebased MAFs and ACMG practice guideline-based pathogenicity of every yielded gene variant. The variants with a minor allele frequency of <0.05 in population databases, such as 1,000 genome, ESP6500, dbSNP, EXAC, and in-house database (MyGenostics), expected to affect protein coding/splicing or present in the Human Gene Mutation Database (HGMD), were included in the analysis. for CNV calling, SAMtools was used to calculate the every-coding-region total bases. The GATK ‘DepthofCoverage’ command was used to obtain the average mean depth of CCDS (Consensus Coding Sequence) regions. R was then used to calculate the ratio of every sample compared with other samples’ mean ratio, and ggplot was used to visualize results. A ratio > 1.4 was assigned as a duplication, and < 0.6 was assigned as a deletion. To reduce false positives, only deletions or duplications of two consecutive exons were identified as true variants.
