# Supplementary material for: Early graft loss due to acute thrombotic microangiopathy accompanied by complement gene variants in living-related kidney transplantation: case series report
Source: BMC Nephrol. 2022 Jul 14;23:249. doi: 10.1186/s12882-022-02868-7 (PMC9284761; doi:10.1186/s12882-022-02868-7)
Supplement: Supplementary file 2 — Additional file 2: Supplementary Table 1. Genes which were sequenced in the study. [file 12882_2022_2868_MOESM2_ESM.doc]

**Additional file 2: Supplementary Table 1.** Genes which were sequenced in the study.

**Supplementary Table 1.** Genes which were sequenced in the study.

| *ACD* | *ACP5* | *ACTB* | *ADA* | *ADA2* | *ADAM17* | *ADAR* | *ADGRE2* |
| --- | --- | --- | --- | --- | --- | --- | --- |
| *AICDA* | *AIRE* | *AK2* | *ALPI* | *AP1S3* | *AP3B1* | *AP3D1* | *APOL1* |
| *ARHGEF1* | *ARPC1B* | *ATM* | *ATP6AP1* | *B2M* | *BACH2* | *BCL10* | *BCL11B* |
| *BLM* | *BLNK* | *BRCA1* | *BRCA2* | *BRIP1* | *BTK* | *C1QA* | *C1QB* |
| *C1QC* | *C1R* | *C1S* | *C2* | *C3* | *C4A* | *C4B* | *C5* |
| *C6* | *C7* | *C8A* | *C8B* | *C8G* | *C9* | *CARD11* | *CARD14* |
| *CARD9* | *CARMIL2* | *CASP10* | *CASP8* | *CCBE1* | *CD19* | *CD247* | *CD27* |
| *CD3D* | *CD3E* | *CD3G* | *CD40* | *CD40LG* | *CD46* | *CD55* | *CD59* |
| *CD70* | *CD79A* | *CD79B* | *CD81* | *CD8A* | *CDCA7* | *CEBPE* | *CFB* |
| *CFD* | *CFH* | *CFHR1* | *CFHR2* | *CFHR3* | *CFHR4* | *CFHR5* | *CFI* |
| *CFP* | *CFTR* | *CHD7* | *CIB1* | *CIITA* | *CLCN7* | *CLEC7A* | *CLPB* |
| *COL7A1* | *COLEC11* | *COPA* | *CORO1A* | *CR2* | *CSF2* | *CSF2RA* | *CSF2RB* |
| *CSF3R* | *CTC1* | *CTLA4* | *CTPS1* | *CTSC* | *CXCR4* | *CYBA* | *CYBB* |
| *CYBC1* | *DBR1* | *DCLRE1B* | *DCLRE1C* | *DDX58* | *DEF6* | *DKC1* | *DNAJC21* |
| *DNASE1L3* | *DNASE2* | *DNMT3B* | *DOCK2* | *DOCK8* | *EFL1* | *ELANE* | *EPG5* |
| *ERBIN* | *ERCC4* | *ERCC6L2* | *EXTL3* | *FAAP24* | *FADD* | *FANCA* | *FANCB* |
| *FANCC* | *FANCD2* | *FANCE* | *FANCF* | *FANCG* | *FANCI* | *FANCL* | *FANCM* |
| *FAS* | *FASLG* | *FAT4* | *FCGR3A* | *FCHO1* | *FCN3* | *FERMT1* | *FERMT3* |
| *FOXN1* | *FOXP3* | *FPR1* | *G6PC3* | *G6PD* | *GATA2* | *GFI1* | *GINS1* |
| *GUCY2C* | *HAVCR2* | *HAX1* | *HELLS* | *HMOX1* | *HPS1* | *HPS4* | *HPS6* |
| *HYOU1* | *ICOS* | *ICOSLG* | *IFIH1* | *IFNAR1* | *IFNAR2* | *IFNG* | *IFNGR1* |
| *IFNGR2* | *IGHM* | *IGKC* | *IGLL1* | *IKBKB* | *IKBKG* | *IKZF1* | *IL10* |
| *IL10RA* | *IL10RB* | *IL12B* | *IL12RB1* | *IL12RB2* | *IL17F* | *IL17RA* | *IL17RC* |
| *IL18BP* | *IL1RN* | *IL21* | *IL21R* | *IL23R* | *IL2RA* | *IL2RB* | *IL2RG* |
| *IL36RN* | *IL6* | *IL6R* | *IL6ST* | *IL7R* | *INO80* | *IRAK1* | *IRAK4* |
| *IRF2BP2* | *IRF3* | *IRF4* | *IRF7* | *IRF8* | *IRF9* | *ISG15* | *ITCH* |
| *ITGB2* | *ITK* | *JAGN1* | *JAK1* | *JAK3* | *KDM6A* | *KMT2A* | *KMT2D* |
| *KRAS* | *LACC1* | *LAMTOR2* | *LAT* | *LCK* | *LIG1* | *LIG4* | *LPIN2* |
| *LRBA* | *LYST* | *MAD2L2* | *MAGT1* | *MALT1* | *MAP3K14* | *MASP1* | *MASP2* |
| *MCM4* | *MEFV* | *MOGS* | *MRE11* | *MRTFA* | *MS4A1* | *MSH6* | *MSN* |
| *MTHFD1* | *MVK* | *MYD88* | *MYSM1* | *NBAS* | *NBN* | *NCF1* | *NCF2* |
| *NCF4* | *NCSTN* | *NFAT5* | *NFE2L2* | *NFKB1* | *NFKB2* | *NFKBIA* | *NHEJ1* |
| *NHP2* | *NLRC4* | *NLRP1* | *NLRP12* | *NLRP3* | *NOD2* | *NOP10* | *NRAS* |
| *NSMCE3* | *OAS1* | *ORAI1* | *OSTM1* | *OTULIN* | *PALB2* | *PARN* | *PEPD* |
| *PGM3* | *PIK3CD* | *PIK3R1* | *PLCG2* | *PLEKHM1* | *PMS2* | *PNP* | *POLA1* |
| *POLD1* | *POLD2* | *POLE* | *POLE2* | *POLR3A* | *POLR3C* | *POLR3F* | *PRF1* |
| *PRKCD* | *PRKDC* | *PSEN1* | *PSENEN* | *PSMB8* | *PSMG2* | *PSTPIP1* | *PTEN* |
| *PTPRC* | *RAB27A* | *RAC2* | *RAD51* | *RAD51C* | *RAG1* | *RAG2* | *RANBP2* |
| *RASGRP1* | *RBCK1* | *REL* | *RELA* | *RELB* | *RFWD3* | *RFX5* | *RFXANK* |
| *RFXAP* | *RHOH* | *RIPK1* | *RMRP* | *RNASEH2A* | *RNASEH2B* | *RNASEH2C* | *RNF168* |
| *RNF31* | *RNU4ATAC* | *RORC* | *RPSA* | *RTEL1* | *SAMD9* | *SAMD9L* | *SAMHD1* |
| *SBDS* | *SEC61A1* | *SEMA3E* | *SERPING1* | *SH2D1A* | *SH3BP2* | *SH3KBP1* | *SHARPIN* |
| *SKIV2L* | *SLC29A3* | *SLC35C1* | *SLC37A4* | *SLC39A7* | *SLC46A1* | *SLC7A7* | *SLX4* |
| *SMARCAL1* | *SMARCD2* | *SNX10* | *SP110* | *SPINK5* | *SPPL2A* | *SRP54* | *SRP72* |
| *STAT1* | *STAT2* | *STAT3* | *STAT5B* | *STIM1* | *STK4* | *STN1* | *STX11* |
| *STXBP2* | *TAP1* | *TAP2* | *TAPBP* | *TAZ* | *TBK1* | *TBX1* | *TCF3* |
| *TCIRG1* | *TCN2* | *TERC* | *TERT* | *TFRC* | *TGFB1* | *TGFBR1* | *TGFBR2* |
| *THBD* | *TICAM1* | *TINF2* | *TIRAP* | *TLR3* | *TMC6* | *TMC8* | *TMEM173* |
| *TNFAIP3* | *TNFRSF11A* | *TNFRSF13B* | *TNFRSF13C* | *TNFRSF1A* | *TNFRSF4* | *TNFRSF9* | *TNFSF11* |
| *TNFSF12* | *TOP2B* | *TP53* | *TPP1* | *TPP2* | *TRAC* | *TRAF3* | *TRAF3IP2* |
| *TREX1* | *TRIM22* | *TRNT1* | *TTC37* | *TTC7A* | *TYK2* | *UBE2T* | *UNC13D* |
| *UNC93B1* | *UNG* | *USB1* | *USP18* | *VPS13B* | *VPS45* | *WAS* | *WDR1* |
| *WIPF1* | *WRAP53* | *XIAP* | *XRCC2* | *ZAP70* | *ZBTB24* | *ZNF341* |  |
